# Selection of Solubility Enhancement Technologies for S-892216, a Novel COVID-19 Drug Candidate

**DOI:** 10.3390/pharmaceutics17121627

**Published:** 2025-12-18

**Authors:** Ryo Ohashi, Shuichi Otake, Tatsuhiko Murata, Ryosuke Watari, Shinpei Yoshida, Mikiko Kitade, Daisuke Kondo, Go Kimura

**Affiliations:** 1 Formulation R&D Laboratory, Pharmaceutical Technology Research Division, Shionogi & Co., Ltd., 1-3 Kuise, Terajima 2-chome, Amagasaki 660-0813, Japan; 2 Laboratory for Drug Discovery and Development, Shionogi & Co., Ltd., 1-1 Futaba-cho 3-chome, Toyonaka 561-0825, Japan

**Keywords:** poorly water-soluble drug, solubility enhancement technology, amorphous solid dispersion, oral solution, stability

## Abstract

**Background/Objectives**: S-892216 is a poorly water-soluble drug developed as a novel oral treatment for COVID-19, although its oral absorption is low. For Phase 1 (Ph1) studies and commercial use, both oral solution and solid dispersion technologies are evaluated to enhance drug solubility. **Methods**: The solubility enhancement technology was selected by considering physicochemical factors such as stability and oral absorption, along with patient and customer acceptability. **Results**: Pharmacokinetics study in rats revealed that both the polyethylene glycol 400 oral solution and polyvinylpyrrolidone-vinyl acetate (PVPVA) amorphous solid dispersion powder suspension showed almost 100% oral bioavailability. Therefore, they can be proposed as clinical formulations for Ph1 studies. PVPVA solid dispersion tablets were developed as a to-be-marketed formulation showed higher bioavailability in dogs than the anhydrous crystal formulation. Additionally, the stability of the developed solid dispersion tablet was acceptable. **Conclusions**: This study demonstrates that multiple solubility enhancement technologies can be adopted for S-892216 development, and amorphous solid dispersion technology was selected for commercialization.

## 1. Introduction

Coronavirus disease 2019 (COVID-19), induced by the novel virus severe acute respiratory syndrome coronavirus 2 (SARS-CoV-2), emerged in late 2019 and rapidly evolved into a global public health emergency [1]. [5-(3-Chloro-4-fluorophenyl)-3-(5-chloropyridin-3-yl)-6-(6,6-difluoro-2-azaspiro [3.3] heptan-2-yl)-2,4-dioxo-3,4-dihydropyrimidin-1(2H)-yl] acetonitrile (denoted as S-892216) is a small-molecule compound developed by Shionogi & Co., Ltd. as a second-generation 3CLpro inhibitor against SARS-CoV-2 [2]. During the drug discovery stage, we found that S-892216 anhydrous crystal exhibits very low water solubility and low oral absorption.

The poor water solubility of drugs limits their oral bioavailability (BA) and presents challenges in the development of oral solid dosage forms. This issue is especially prevalent among new drug candidates, with approximately 40% of marketed drugs and 90% of new chemical entities exhibiting poor water solubility [3,4]. However, there is no universal technique for formulating these drugs. Formulation studies using commercially established approaches to improve the dissolution and thus bioavailability of these drugs are desirable for the pharmaceutical industry to expedite the development stage.

There are some strategies to enhance the solubility and absorption of poorly water-soluble drugs [5,6]. One of the standard approaches is the crystal engineering of drug substances, namely the formation of salts, solvates, and co-crystals [7,8,9,10,11]. By integrating micronization with crystal engineering, for example, by using a jet mill [12] it becomes possible to manufacture tablets or capsules using conventional manufacturing processes [13]. When crystal engineering fails, formulation-based approaches may be explored to enhance solubility. Popular formulations include amorphous solid dispersions, which stabilizes the amorphous form of the drug [14,15,16,17], and oral solutions such as lipid-based formulations, which maintains the dissolved form of the drug [18,19,20]. These technologies have been in practical use for more than a decade, supported by numerous research findings and multiple commercial products [17].

Pharmaceutical companies consider multiple factors beyond just improving oral absorption in the selection of appropriate solubility enhancement technologies. It is necessary to conduct clinical studies in a sequential manner during drug development. Phase 1 clinical studies often employ assessments of absorption, distribution, metabolism, and excretion (ADME) [21,22]. Pharmaceutical companies sometimes need to develop a dose-flexible formulation for human ADME studies only [23,24]. In the development of to-be-marketed formulations, the shelf-life of the product, which is determined from stability data, impacts supply chain costs and customer satisfaction [25,26]. Amorphous solid dispersions and oral solutions are promising formulations that enhance solubility, although they tend to be chemically unstable compared with the crystalline form [27,28,29,30]. From another perspective, the dosage form can affect the ease of ingestion, namely swallowing, which can in turn impact patient acceptability [31,32,33]. Although numerous research studies on solubility enhancement technologies have been reported, to the best of our knowledge, few studies have discussed the industrial applicability of these technologies considering the factors mentioned above. Here, we describe the approach by which we evaluated the ADME of various formulations of S-892216 and developed tablets for the clinical development program of S-892216.

By comparing multiple formulation approaches, including PEG400-based solution and PVPVA-based solid dispersion, the research demonstrated that solubility enhancement technologies can significantly improve bioavailability. The findings revealed the value of a flexible, stage-specific approach to formulation design, offering practical insights into the development of challenging drug candidates.

## 2. Materials and Methods

### 2.1. Materials

**Figure 1:**
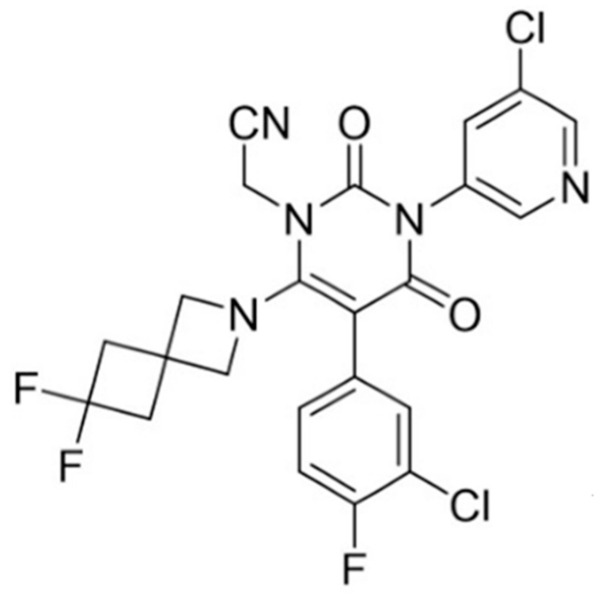
Chemical structure of S-892216 drug substance [2].

S-892216 drug substance was designed by Shionogi & Co., Ltd. (Osaka, Japan). The chemical structure of S-892216 is shown in Figure 1 [2]. The molecular formula and molecular weight of S-892216 are C23H16Cl2F3N5O2 and 522.31, respectively. S-892216 was synthesized as an anhydrous crystal, and its powder X-ray diffraction (PXRD) pattern is presented in Figure S1. Polyethylene glycol 400 (PEG 400) (Kollisolv PEG 400, BASF, Ludwigshafen, Germany), propylene glycol (PG) (Kollisolv PG, BASF) were used as solvents for oral solution development to improve drug dissolution. Ascorbic acid (DSM Nutritional Products AG, Kaiseraugst, Switzerland) was included as an antioxidant to prevent oxidative degradation. Polyvinylpyrrolidone-vinyl acetate (PVPVA) (Plasdone S-630, Ashland Inc., Lexington, KY, USA) and hydroxypropyl methylcellulose acetate succinate (HMPCAS) (HPMCAS-LF, Shin-Etsu Chemical Co., Ltd., Tokyo, Japan) were used as polymers to stabilize the amorphous form and enhance dissolution in solid dispersion systems. They were chosen because they are widely used pharmaceutical grade polymers and authors have prior experience with these materials in drug product development. Mannitol (Pearlitol 200SD, Roquette Freres, Lestrem, France) and microcrystalline cellulose (Ceolus PH-102, Asahi Kasei Corporation, Tokyo, Japan) were fillers to provide tablet strength and compressibility. Croscarmellose sodium (Ac-Di-Sol^®^ SD-711, DuPont, Wilmington, DE, USA) was used as the disintegrant to ensure rapid tablet disintegration. Magnesium stearate (NF Hyqual, Mallinckrodt Inc., Saint Louis, MO, USA) and sodium stearyl fumarate (PRUV, JRS PHARMA, Rosenberg, Germany) were used as the lubricant to prevent sticking. The coating agent was a premix product containing hypromellose, talc, red ferric oxide, and yellow ferric oxide (Opadry, Colorcon Inc., West Point, PA, USA) for light protection and appearance. All excipients used are commonly employed in solid dispersion research and conventional tablet design, ensuring that the formulation approach aligns with established pharmaceutical practices. All other chemicals and solvents were commercially available analytical-grade reagents.

### 2.2. Preparation of Samples

#### 2.2.1. PEG 400 Solution

The component and composition of S-892216 oral solution are shown in Table 1. First, 4.7 g of PVPVA and 1.8 g of ascorbic acid were added and dissolved in 160 g of PEG 400. Then, 25 mg of S-892216 anhydrous crystal was dissolved in 9618.8 mg of the PEG 400 solution. Finally, 5.0 g of drug-containing solution and 4.3 g of PG were mixed using a stirrer to obtain the oral solution.

#### 2.2.2. Amorphous Solid Dispersion Powder

Polymer and the S-892216 drug substance were dissolved in acetone at 4 g/batch. Acetone was chosen because both the drug and the polymers exhibited good solubility in this solvent. Our experimental results confirmed that the drug and polymers could dissolve at concentrations of approximately 10% *w*/*w* in acetone, which is similar with literature recommendations [34], suggesting that 5% *w*/*w* is preferable for spray-drying processes. Drug-to-polymer ratio was based on the formulation design described in Section 3.2.1, where the drug loading was set according to screening results. After confirming that they were completely dissolved, the solid dispersion powder was prepared using a spray dryer (Spray Dryer B-290 Nihon BUCHI K.K., Tokyo, Japan). The conditions for production were an inlet temperature of 90 °C, liquid delivery pump at 20%.

#### 2.2.3. Anhydrous Crystal Capsule

The component and composition of samples are shown in Table 2. S-892216 drug substance, mannitol, microcrystalline cellulose, croscarmellose sodium, and magnesium stearate were mixed using a mortar and pestle. The drug concentration (20% *w*/*w*) was selected based on author’s formulation development experience to ensure that the capsule size remained suitable for administration to dogs. The API exhibited strong adhesion and poor flowability, making direct capsule filling impractical. Therefore, we decided to conduct granulation to improve handling. Among available methods, wet granulation and dry granulation were considered. However, because the impact of water on the API was unknown, we selected dry granulation to avoid potential stability risks. The mixture was sieved through a 20-mesh screen and, subsequently, compressed for dry granulation (slugging) using a single-station tablet press (Ichihashi Seiki Co., Ltd., Kyoto, Japan) with a flat-faced punch (diameter = 15 mm). The slug weight was adjusted to 500 mg and compressed at the pressure of 12 kN. The slugs were sized using a 20-mesh screen. Magnesium stearate was added to the milled granules. The mixture was lubricated by hand shaking in a glass vial. The final blend was filled into empty capsules by hand.

#### 2.2.4. Amorphous Solid Dispersion Uncoated Tablet

The component and composition of samples are shown in Table 3. S-892216 amorphous solid dispersion, mannitol, microcrystalline cellulose, croscarmellose sodium, and magnesium stearate were mixed using a mortar and pestle. The concentration of the solid dispersion powder in the tablet was set at 40% *w*/*w* (equivalent to 10% *w*/*w* as drug) to minimize tablet size while maintaining acceptable disintegration properties. Based on author’s experience, higher concentrations of solid dispersion powder in the tablets could lead to polymer gelation and impaired disintegration. Therefore, concentrations above this level were not tested. For solid dispersion formulations, dry processes are generally preferred to prevent moisture-induced crystallization [35]. While both direct compression and dry granulation are possible, direct compression carries a high risk of manufacturing issues when powder flowability is poor. Our formulation contained 40% solid dispersion powder, which could compromise flowability; therefore, we selected dry granulation from the outset to ensure robust manufacturability. The mixture was sieved through a 20-mesh screen and, subsequently, compressed for dry granulation (slugging) using a single-station tablet press (Ichihashi Seiki Co., Ltd., Kyoto, Japan) with a flat-faced punch (diameter = 15 mm). The slug weight was adjusted to 500 mg and compressed at the pressure of 12 kN. The slugs were sized using a 20-mesh screen. Magnesium stearate was added to the milled granules. The mixture was lubricated by hand shaking in a glass vial. The final blend was compressed using a single-station tablet press with a round-shaped punch (diameter = 8 mm).

#### 2.2.5. Amorphous Solid Dispersion-Coated Tablet

First, 7.5 kg of S-892216 drug substance and 22.5 kg of PVPVA were dissolved in 270 kg of acetone. After confirming that they were completely dissolved, the solid dispersion powder was prepared using a production-scale spray dryer (PHARMA-SD^®^ PSD-4, GEA, Dusseldorf, Germany). The conditions for production were an outlet temperature of 50 °C, feed rate of 80 kg/h, and spray pressure of 2.5 bar. Obtained solid dispersion powder was dried in a vacuum dryer for 37 h. The batch size of tablet manufacturing was 20 kg/batch. S-892216 amorphous solid dispersion, mannitol, microcrystalline cellulose, croscarmellose sodium and sodium stearyl fumarate were mixed using a blender for 15 min. The mixture was sieved through a screening mill with a 1.6 mm opening screen (Quadro Comil 197S, Powrex Corporation, Hyogo, Japan) and, subsequently, dry granulated using a roller compactor at the pressure of 3–7 MPa. Ribbons were sized using a 20-mesh screen. Sodium stearyl fumarate was added to the milled granules. The mixture was lubricated for 3 min using a 60 L V-blender. The final blend was compressed using a rotary tablet press (VIRGO, Kikusui Seisakusho Ltd., Kyoto, Japan) with an oval-shaped punch (long axis = 14.0 mm, short axis = 7.3 mm). Based on our pharmaceutical development experience, oval-shaped punches were selected to improve swallowability for patients, particularly for tablets weighing approximately 400 mg, where oval shapes are generally preferred over round shapes for ease of ingestion. Film coating was performed using a pan-coating machine (HC-FZ-80F, Freund Industrial Co., Ltd., Tokyo, Japan) to improve photostability (discussed in the Supplemental Materials, Section S.10.). The coating agent was a premix product containing hypromellose, talc, red ferric oxide, and yellow ferric oxide (Opadry, Colorcon Inc., West Point, PA, USA). The conditions for production were an inlet temperature of 60 °C, feed rate of 40–80 g/min, and spray pressure of 0.4 MPa.

### 2.3. Pharmacokinetics of S-892216 in Rats and Dogs

#### 2.3.1. Animals

Male Sprague–Dawley (Crl:CD(SD), Jackson Laboratory Japan Inc., Kanagawa, Japan) were used for experiments at 8 weeks of age. Male Marshall Beagle dogs (Marshall BioResources Japan Inc., Ibaraki, Japan) were used for experiments at 1 year of age.

#### 2.3.2. Rat Pharmacokinetic Study

PEG 400 solution formulation of S-892216 anhydrous crystal was orally administered to rats (*n* = 3) at 1 mg/kg (weight = approximately 300 g) under a fasted condition, and then 2 mL/kg of water for injection was orally administered. The oral solution formulation development is discussed in Section 3.2.1. Blood samples were serially collected at 0.5, 1, 2, 4, 8, and 24 h after dosing and centrifuged to obtain plasma samples. S-892216 amorphous solid dispersion powder was suspended in 0.5% (*w*/*v*) methylcellulose 400 solution (0.5 *w*/*v*% Methyl Cellulose 400 Solution Sterilized, FUJIFILM Wako Pure Chemical Corporation, Osaka, Japan) at a concentration of 4 mg per 2 mL (equivalent to 1 mg as drug), and then orally administered to rats (*n* = 3) at 1 mg/kg under a fasted condition. Methylcellulose was used to ensure uniform dispersion of the solid dispersion powder in the suspension. Blood samples were serially collected from the jugular vein up to 24 h after dosing and centrifuged to obtain plasma samples. The plasma concentration of S-892216 was determined using liquid chromatography with tandem mass spectrometry (LC/MS/MS). The MS systems were SCIEX Triple Quad 5500, 6500, and 6500+ (AB Sciex LLC, Framingham, MA, USA) and Xevo TQ-XS (Waters Corporation, Milford, MA, USA). After extraction with acetonitrile, samples were injected into a YMC-Triart C18 column (3 mm, 2.1 mm i.d. × 50 mm, YMC Co., Ltd., Kyoto, Japan) and eluted from the column using a gradient program (representative), with mobile phase A consisting of 0.1% (*v*/*v*) formic acid in water and mobile phase B consisting of acetonitrile, as summarized in Table S1. The flow rate was 0.75 mL/min. For detection using electrospray ionization in the positive ion mode, the multiple reaction monitoring precursor/product ion transition was m/z 522/368.

#### 2.3.3. Dog Pharmacokinetic Study

S-892216 anhydrous crystal capsules or solid dispersion tablets were orally administered to dogs (*n* = 3) at 3 mg/kg under the fed condition. After administration, 25 mL of water for injection was orally administered. Blood samples were serially collected from the forelimb vein up to 48 h after dosing and centrifuged to obtain plasma samples. The plasma concentration of S-892216 was determined using LC/MS/MS (SCIEX Triple Quad 6500 and SCIEX Triple Quad 6500+). After extraction with acetonitrile, samples were injected into a YMC-Triart C18 column (3 mm, 2.1 × 50 mm) and eluted from the column using a gradient program (representative), with mobile phase A consisting of 0.1% formic acid in water and mobile phase B consisting of acetonitrile, as summarized in Tables S2 and S3. The flow rate was 0.75 mL/min. The multiple reaction monitoring precursor/product ion transition was m/z 522/368.

#### 2.3.4. Pharmacokinetic Analysis

The maximum plasma concentration (C_max_), time to maximum plasma concentration (T_max_), and area under the plasma concentration–time curve (AUC) were calculated by non-compartmental analysis. In addition, BA after an oral administration was calculated using Equation (1):

BA% = (AUC_po_/Dose_po_)/(AUC_iv_/Dose_iv_) × 100 (1)

where subscripts iv and po denote intravenous and oral administration, respectively. The intravenous administration study described in the Supplementary Materials involved *n* = 2 rats and *n* = 4 dogs under fed conditions. Pharmacokinetic data of S-892216 in dogs and rats after a single intravenous administration of S-892216 anhydrous crystal at 0.1 mg/kg under the fed condition are summarized in Table S4 (rats) and Table S5 (dogs), and plasma concentration profiles are shown in Figure S2 (rats) and Figure S3 (dogs).

### 2.4. Evaluation of Degradation Products Level

S-892216 solid dispersion tablet was transferred to a volumetric flask, and acetonitrile/water (1:1) was added. The solution was sonicated for 15 min. The solution volume was adjusted to 50 mL by adding acetonitrile/water (1:1), and the solution was filtered through a 0.45 µm membrane filter. The absorbance of the solution at the wavelength of 247 nm was measured using a UV–HPLC system (ACQUITY UPLC H-Class, Waters Corporation, Milford, MA, USA) equipped with a reverse-phase ODS column (ACQUITY UPLC BEH C18, 1.7 µm, 2.1 × 100 mm, Waters Corporation). The column temperature was maintained at 40 °C. The mobile phase consisted of 0.1% formic acid and acetonitrile. Separation was achieved in 39.5 min using the gradient program summarized in Table S6. The flow rate was 0.3 mL/min throughout the run. The injection volume was 4 µL. The degradation product level was calculated as a percentage (%) of the total area of all peaks in the chromatogram, which was set to 100%.

### 2.5. Dissolution Testing

Dissolution testing was performed using a dissolution apparatus at 50 rpm and 37 °C, following the paddle method. The dissolution medium was the 2nd fluid for dissolution test defined in the Japanese Pharmacopoeia, which is a mixture of phosphate buffer solution (pH 6.8) and water in a 1:1 ratio. The phosphate buffer solution contains 3.40 g of potassium dihydrogen phosphate and 3.55 g of anhydrous disodium hydrogen phosphate in water to make 1000 mL. The pH of the dissolution medium is 6.9–7.0. First, 10 mL of the sample was collected at predefined time points and filtered through a 0.45 µm filter. Then, the absorbance of the filtrate at the wavelength of 299 nm was measured using a UV–HPLC system (ACQUITY UPLC H-Class, Waters Corporation) equipped with a reverse-phase ODS column (X Bridge C18, 3.5 µm, 4.6 mm × 150 mm, Waters Corporation). The column temperature was maintained at 40 °C, and the mobile phase (0.1% formic acid/acetonitrile, 53/47, *v*/*v*) was delivered at the flow rate of 1.0 mL/min. The concentration of the sample solution was determined by comparing the area of the S-892216 peak in the sample solution to that of a standard solution with a known concentration.

## 3. Results and Discussion

### 3.1. S-892216 Anhydrous Crystal

**Figure 2:**
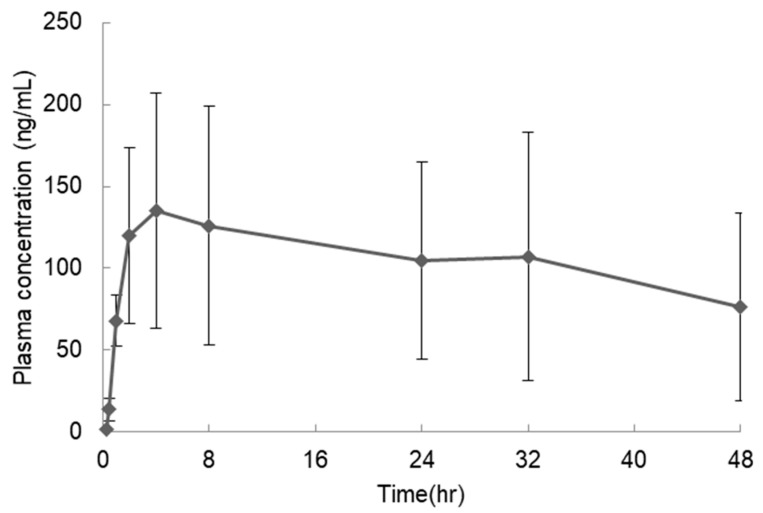
Plasma concentration profiles of S-892216 in dogs after oral administration of S-892216 anhydrate crystalline (3 mg/kg) under the fed condition. Each symbol represents the mean ± SD of 3 dogs.

S-892216 anhydrous crystal exhibited extremely low water solubility, ranging from 0.61 to 1.08 µg/mL, under all pH conditions (Table 4). These values are below the solubility threshold of 10 µg/mL, which is the absorption criterion for a drug orally administered at the dose of 1 mg/kg, as stated by Huang et al. [36], potentially hindering absorption. The BA of S-892216 anhydrous crystal capsules orally administered to dogs was 22.2% (Figure 2 and Table 5). This value is also below the target BA of 70% or more, which is defined as high absorption [37]. Low BA can present serious problems, such as increased dose requirement, absorption variability, and unexpected dose–response relationships. Therefore, we decided to pursue formulation approaches to enhance drug solubility and oral absorption.

### 3.2. Formulation Development for Human ADME Studies

First-in-human studies represent the first step in clinical development [38]. They typically include ADME studies, such as single ascending dose (SAD) studies. SAD studies often require formulations that can be administered at both low and high doses to establish plasma drug concentration correlations [39]. Simplified formulations that allow for dosing flexibility and require less time to develop are often used instead of the to-be-marketed formulation. Dosage forms such as oral solutions, powders (including powders for oral suspensions), and capsules are preferred owing to their dosing flexibility [40,41]. To develop a formulation suitable for human ADME studies, we evaluated the effectiveness of known solubility enhancement approaches, namely developing oral solutions and amorphous solid dispersions of S-892216.

#### 3.2.1. Oral Solution and Solid Dispersion Powder Development

Developing oral solutions is a popular solubility enhancement approach. In the development of oral solutions, determining the solubility of the drug in various solvents is an important stage [42]. As a result of screening various solvents, we found that PEG 400 exhibited the highest solubility at 15–20 mg/mL (Table S7). Therefore, PEG 400 oral solution of S-892216 was proposed for human ADME studies. Oral solutions are a convenient dosage form for human ADME studies owing to advantages such as (1) the possibility of low dose administration because there are no concerns about content uniformity and (2) the flexibility of dose adjustment based on the amount of liquid. The drug concentration was set at approximately 0.1% *w*/*w*, which is well below the saturation solubility (about 1% *w*/*w*), to ensure that no precipitation of the API occurs during administration. We added ascorbic acid as an antioxidant to prevent the degradation of S-892216 in PEG 400. Details of antioxidant selection are provided in Tables S8 and S9. PG was added to adjust the viscosity.

**Figure 3:**
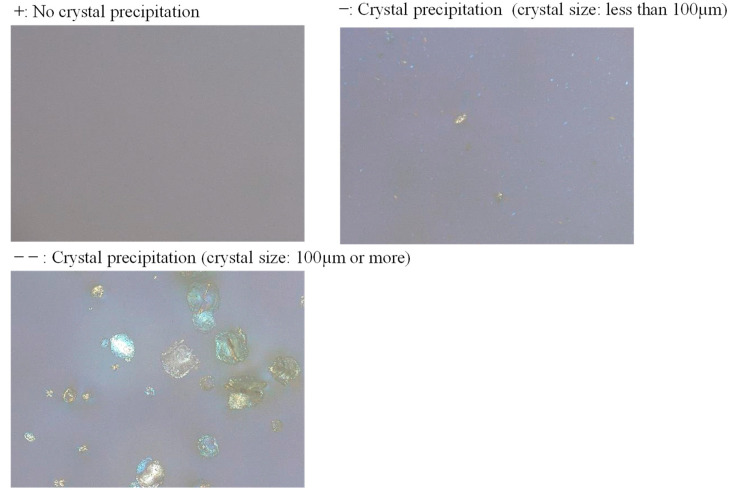
Observation under a polarized optical microscope (×100 magnification, model BX50-33P, Olympus Corporation, Tokyo, Japan).

Developing amorphous solid dispersions is another popular approach to enhance solubility, supported by over 20 commercially available products based on solid dispersions [43]. Solid dispersions are commonly manufactured by spray drying and hot melt extrusion (HME) [44,45]. HME becomes challenging when the melting point of the drug exceeds 220 °C [46]. Because the melting point of S-892216 anhydrous crystal is 243 °C, employing HME likely presents high risks. However, we found that, at the concentration of 10% *w*/*w*, S-892216 drug substance dissolved in acetone, a commonly used solvent for spray drying. Therefore, we chose spray drying to manufacture solid dispersions. To select a suitable polymer as the base for the solid dispersion, a polymer solution was prepared using a small amount of S-892216 drug substance. We then looked for crystal precipitation on a glass slide. The polymer candidates considered were the vinyl polymer of PVPVA and povidone, the cellulose polymer of hypromellose acetate succinate, hypromellose phthalate, and hydroxypropyl cellulose, and the acrylic polymer of methacrylic acid copolymer. S-892216 was dissolved in acetone or a mixture of acetone and ethanol to the concentration of 100 mg/mL and then dropped onto a glass slide. We used polarized microscopy to detect precipitated crystals (1) immediately after applying the solution, (2) after 1 week of storage at 40 °C 75% RH, and (3) after 1 week of storage at 60 °C under uncontrolled humidity (ambient RH) in a stability chamber (model CRH-220, ESPEC CORP., Osaka, Japan). Examination was conducted with the following concentrations of S-892216 in the polymer solution: 10%, 25%, and 50% *w*/*w*. The results are shown in Table 6 and Figure 3. Observation using a microscope revealed that there was no precipitation of coarse crystals larger than 100 µm when PVPVA, PVP, HPMC AS-MF, or HPMC AS-LF was used at the drug substance concentration of 25%. As shown in Table 6, drug loadings of 10%, 25%, and 50% in the solid dispersion exhibited no significant differences in precipitation tendency. Therefore, the mid-level concentration of 25% *w*/*w* was selected for further development.

After selecting the polymer candidate and drug content, we prepared solid dispersion powders with various polymers using a spray dryer. The drug substance content of the solid dispersion powder was 25% *w*/*w*.

**Figure 4:**
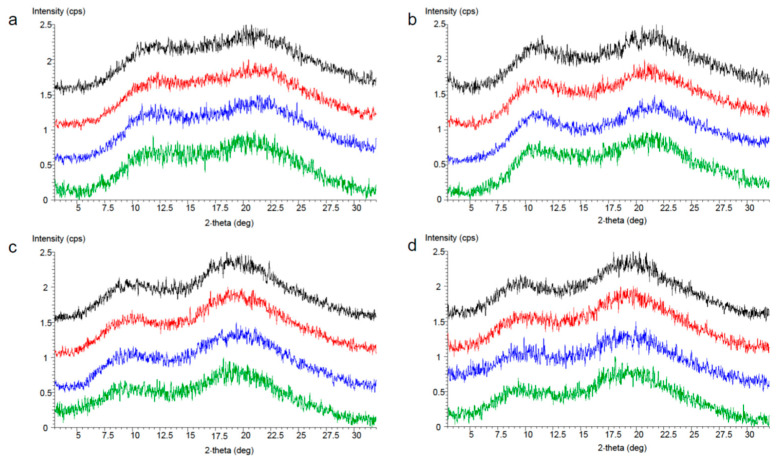
PXRD evaluation of the stability of solid dispersion powder of PVPVA (**a**), PVP (**b**), HPMCAS-MF (**c**) and HPMCAS-LF (**d**). Black: 60 °C, closed glass bottle, 1 week; red: 40 °C 75% RH, closed glass bottle, 1 week; blue: 40 °C 75% RH, open bottle, 1 week; green: time zero.

The crystal form of the obtained solid dispersion powder was evaluated using PXRD. The results are shown in Figure 4. PXRD analysis revealed that solid dispersion powders, which were produced using all polymer additives, remained amorphous after storage (1) at 60 °C in a closed environment for one week and (2) at 40 °C75% RH in both open and closed conditions for one week. Consequently, it was feasible to prepare solid dispersion powder with any of these polymers owing to their stability.

#### 3.2.2. Evaluation of Pharmacokinetics

**Figure 5:**
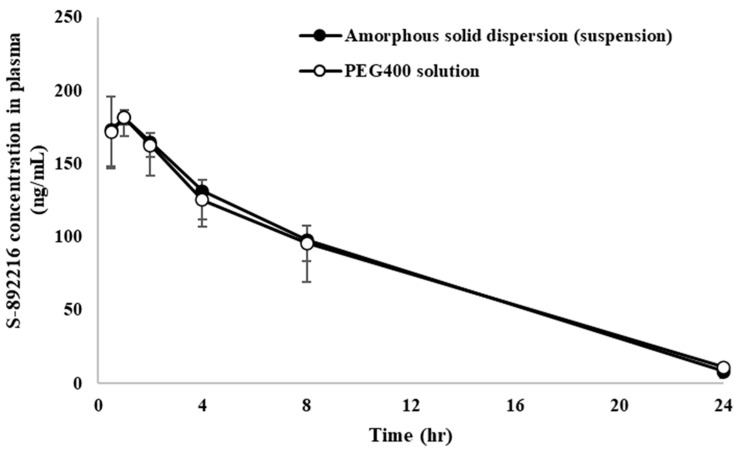
Concentration profiles of S-892216 after a single oral administration of PEG400 solution of S-892216 anhydrate crystal or S-892216 amorphous solid dispersion (1 mg/kg). Each symbol represents the mean ± SD of 3 rats.

PEG 400 oral solution and PVPVA solid dispersion suspension were orally administered to rats under fasted conditions at doses of 1 mg/kg. The results of the rat pharmacokinetic study are shown in Figure 5 and Table 7. The individual plasma concentration–time data for both formulations are provided in Tables S10 and S11. Both the oral solution and solid dispersion suspension was efficient, with BA approaching 100%. Thus, both the oral solution and solid dispersion suspension are solubility enhanced formulations that can be used for human ADME studies.

Here, we discuss the selection criteria for the two formulations. Solid dispersions can be developed as typical dosage forms of tablets or capsules, which can be proposed as to-be-marketed oral formulations. The developed solid dispersion powder contains the drug at the concentration of 25% *w*/*w*, and thus the final concentration of the drug in tablets or capsules is approximately 10% *w*/*w*. Although we can prepare soft gel capsules using the developed oral solution, their drug concentration is just 0.13% *w*/*w* owing to the low solubility of the drug, which makes these capsules bigger than the solid dispersion formulations. The oral solution has other applications. For example, in human mass balance studies commonly performed during drug development, radioactively labeled drug substances are used to evaluate human ADME [21,47]. However, creating a solid dispersion using labeled drug substances might be challenging owing to facility limitations. In this scenario, the oral solution may be helpful to overcome this limitation. Additionally, because the oral solution does not pose any risk in terms of content uniformity, it can also be employed in micro-dosing studies [48,49]. In summary, we believe that developing multiple formulations that enhance absorption will increase the flexibility of ADME studies. This would facilitate rapid and efficient development of S-892216.

### 3.3. Development of To-Be-Marketed Formulation

As of December 2024, the dosage forms of medicines for COVID-19 treatment are tablets and capsules [50,51,52]. These unit-dose solid dosage forms offer several advantages, including ease of storage, portability, ease of administration, and accuracy in dosing [53]. Limenh et al. found that participants in a clinical study preferred tablets over capsules (42.4% vs. 19.9%) [54]. Therefore, we selected tablets as the to-be-marketed formulation of S-892216. On the basis of previous results, we employed spray drying to enhance drug solubility and dry granulation to prepare the to-be-marketed formulation. Dry granulation is a common process in the manufacture of solid dispersion formulations.

#### 3.3.1. Polymer Selection for Solid Dispersion Tablet Development

**Figure 6:**
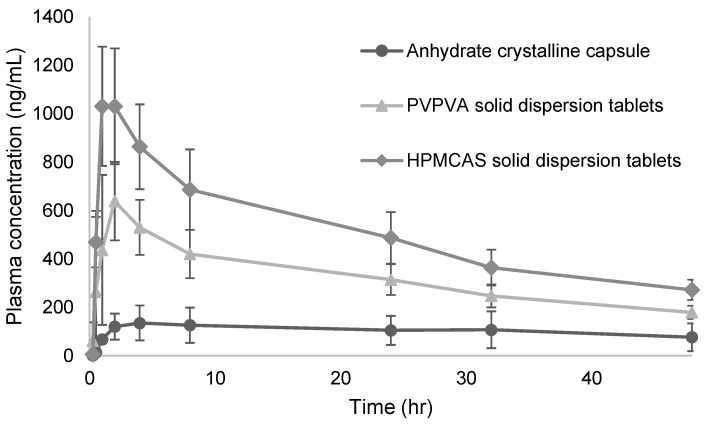
Plasma concentration profiles of S-892216 in dogs after a single oral administration of S-892216 solid dispersion tablets (3 mg/kg) under the fed condition. Each symbol represents the mean ± SD of 3 dogs.

We prepared uncoated tablets using solid dispersion powders with PVPVA and HPMCAS (Table 2). Both polymers have a proven track record of use in commercial products [17]. We evaluated both polymers from the perspective of absorption and tablet stability. The dog pharmacokinetic study was conducted using solid dispersion tablets at the dose of 3 mg/kg. The results of the dog pharmacokinetic study are shown in Figure 6 and Table 8. According to the AUC, the absorption of solid dispersion tablets was approximately 4 to 6 times greater than that of anhydrous crystal capsules. Moreover, the absorption of HPMCAS solid dispersion tablets was higher than that of PVPVA tablets. This finding aligns with the results of dissolution testing (Figure S4), suggesting that the solubility of the HPMCAS solid dispersion is higher than that of the PVPVA solid dispersion, potentially leading to enhanced absorption.

Next, we conducted a short-term stability study using solid dispersion tablets. After two weeks of storage at 60 °C, the degradation product level of PVPVA solid dispersion tablets (0.11%) was lower than that of HPMCAS solid dispersion tablets (0.44%) as shown in Table 9. This difference likely arises from the potential incompatibility between the drug and polymer. On the basis of these results, we selected PVPVA solid dispersion tablets for further development because of its enhanced absorption and high stability.

#### 3.3.2. Stability Study of Developed Solid Dispersion Tablets

**Figure 7:**
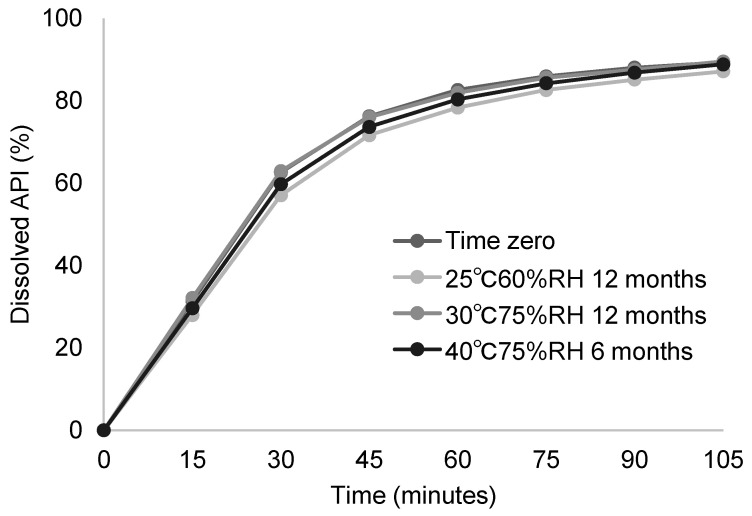
Dissolution of PVPVA solid dispersion tablets in the stability testing (*n* = 6 for time zero, *n* = 3 for others).

The stability of the developed PVPVA solid dispersion tablets (Table 10) was evaluated under long-term, intermediate, and accelerated conditions for 12 months, 12 months and 6 months, respectively. The selection of film coating is discussed in the Supplemental Materials (Section S.10., Tables S12 and S13). The results of dissolution testing demonstrated that the formulation maintained supersaturation throughout the stability testing (Figure 7), confirming that the amorphous form was stabilized through the employed solid dispersion technology. The results of degradation product analysis were also acceptable (Table 11). On the basis of the above findings, we adopted this formulation for late-stage clinical studies and to-be-marketed product manufacturing.

In the development of poorly water-soluble compounds, it is necessary to consider whether solubility enhancement technologies can be applied, as well as factors such as dosage form selection and stability. In this study, we found that both the oral solution and solid dispersion are applicable to enhance drug solubility for human ADME studies and that the solid dispersion is better than the oral solution for preparing the to-be-marketed formulation. We hope that the findings of this study will advance the development of S-892216 and contribute to improving the quality of life for patients suffering from COVID-19 around the world.

## 4. Conclusions

We succeeded in developing formulations of poorly water-soluble drug S-892216 by applying solubility enhancement technologies. Oral solution and solid dispersion suspension were developed as formulations for human ADME studies, and their efficient absorption was confirmed through a rat pharmacokinetic study. In developing solid dispersion-coated tablets as the to-be-marketed formulation, PVPVA was selected as a suitable polymer from the results of both the dog pharmacokinetic study and stability testing. Our findings indicate that the to-be-marketed formulation will facilitate rapid and efficient evaluation of S-892216 through human ADME studies as well as large-scale clinical studies.

## Figures and Tables

**Table 1:** Component and composition of S-892216 oral solution and solid dispersion suspension.

Component	Composition (mg)	Composition (%*w*/*w*)
S-892216 drug substance	0.30	0.14
PVPVA	3.26	1.51
Ascorbic acid	1.25	0.58
PEG 400	110.9	51.53
Propylene glycol	99.5	46.24
Total	215.2	100.0

**Table 2:** Component and composition of anhydrous crystal capsules.

Component	Composition (mg)
S-892216 drug substance	30.0
Mannitol	54.8
Microcrystalline cellulose	54.8
Croscarmellose sodium	7.5
Magnesium stearate	3.0
Total (mg)	150.0
Hypromellose capsule	1 unit

**Table 3:** Component and composition of solid dispersion tablets.

Component	Composition (mg)	
Formulation	PVPVA solid dispersion tablet	HPMCAS solid dispersion tablet
S-892216-PVPVA Solid dispersion (as S-892216)	120.0 (30.0)	-
S-892216-HMPCAS-LF Solid dispersion (as S-892216)	-	120.0 (30.0)
Mannitol	72.0	72.0
Microcrystalline cellulose	72.0	72.0
Croscarmellose sodium	30.0	30.0
Magnesium stearate	6.0	6.0
Total (mg)	300.0	300.0

**Table 4:** Solubility of S-892216 anhydrous crystal.

Solvent ^1^	Solubility (µg/mL)
Purified water	0.71
JP-1 fluid (pH 1.2)	1.08
McIlvaine buffer (pH 4.0)	0.64
JP-2 fluid (pH 6.8)	0.61

^1^ JP-1 fluid (pH 1.2) was prepared by dissolving 2.0 g of sodium chloride in 7.0 mL of hydrochloric acid and adjusting the volume to 1000 mL with water. JP-2 is a mixture of phosphate buffer solution (pH 6.8) and water (1:1).

**Table 5:** Pharmacokinetic parameters of S-892216 in dogs after a single oral administration of S-892216 anhydrate crystalline under the fed condition.

Dose (mg/kg)	C_max_ (ng/mL)	T_max_ (h)	AUC_inf_ (ng·h/mL)	BA (%)
3	152 ± 71	12.7 ± 16.8	6500 ± 4120 ^1^	22.2 ± 14.1

Data represent the mean ± SD of 3 dogs. ^1^ In 1 of the 3 dogs, an appropriate elimination phase could not be achieved owing to slow absorption (T_max_ = 32 h), and therefore the AUC up to 48 h after dosing was used in this case.

**Table 6:** Results of polymer screening for solid dispersion with different drug concentration.

Storage Period		Solvent (Acetone: Ethanol)	Time Zero			40 °C75%RH 1 Week			60 °C 1 Week		
Drug Concentration in Solid Dispersion (% *w*/*w*)			10	25	50	10	25	50	10	25	50
Polymer	PVPVA	100:0	+	+	−	+	+	+	+	+	+
	PVP	80:20	+	+	+	+	+	+	+	+	+
	HPMC AS-MF	20:1	−	−	−−	−	−	−−	−	−	−−
	HPMC AS-LF	20:1	−	−	−−	−	−−	−−	−	−−	−−
	HPMCP	8:2	−	−−	−−	−	−−	−−	−	−−	−−
	HPC SL	8:2	−	−−	−−	−−	−−	−−	−−	−−	−−
	EUDRAGIT L100	1:1	+	−	−	+	−−	−−	+	−−	−−

+ No crystal precipitation; − Crystal precipitation (crystal size less than 100 µm); −− Crystal precipitation (crystal size greater than 100 µm).

**Table 7:** Pharmacokinetic parameters of S-892216 in rats after a single oral administration of PEG 400 solution or solid dispersion suspension (1 mg/kg).

Formulation	Dose	Feeding	C_max_	T_max_	AUC_inf_	BA
			(ng/mL)	(h)	(ng·h/mL)	(%)
PEG 400 solution	1 mg/kg	Fasted	186 ± 12	0.833 ± 0.289	1972 ± 157	101 ± 8
PVPVA solid dispersion suspension		Fasted	182 ± 13	0.83 ± 0.29	1970 ± 480	110 ± 27

Data represent the mean ± SD of 3 rats.

**Table 8:** Pharmacokinetic parameters of S-892216 in dogs after a single oral administration of S-892216 solid dispersion tablets (3 mg/kg) under the fed condition.

Formulation	C_max_	T_max_	AUC_inf_	BA
	(ng/mL)	(h)	(ng·h/mL)	(%)
Anhydrous crystal capsules	152 ± 71	12.7 ± 16.8	6500 ± 4120	22.2 ± 14.1
HPMCAS solid dispersion tablets	1040 ± 241	1.67 ± 0.58	36,200 ± 5300	123.6 ± 18.0
PVPVA solid dispersion tablets	697 ± 177	1.67 ± 0.58	23,200 ± 3300	79.2 ± 11.1

Data represent the mean ± SD of 3 dogs. In 1 of the 3 dogs, an appropriate elimination phase could not be achieved owing to slow absorption (T_max_ = 32 h), and therefore the AUC up to 48 h after dosing was used in this case.

**Table 9:** Degradation of solid dispersion tablets stored in a sealed glass vial at 60 °C for 2 weeks.

Storage Period	Amount of Degradation Product (Individual Max)	
	1 Week	2 Weeks
PVPVA solid dispersion tablet	0.08%	0.11%
HPMCAS solid dispersion tablet	0.32%	0.44%

**Table 10:** Solid dispersion-coated tablets for stability testing.

Component	Composition (mg)
S-892216 PVPVA Solid dispersion (as S-892216)	160.0 (40.0)
Mannitol	148.8
Microcrystalline cellulose	37.2
Croscarmellose sodium	40.0
Colloidal silicon dioxide	2.0
Sodium stearyl fumarate	12.0
Subtotal	400.0
Coating agent (Hypromellose) (Talc) (Red Ferric Oxide) (Yellow Ferric Oxide)	16.0 (70.330% *w*/*w*) (28.966% *w*/*w*) (0.352% *w*/*w*) (0.352% *w*/*w*)
Total	416.0

**Table 11:** Degradation Products levels of solid dispersion tablets in the stability testing.

Storage Condition and Period	Amount of Degradation Product (%, Individual Max)			Total (%)
	Degradation Product A	Degradation Product B	Others	
Time zero	<0.10	0.01	<0.10	0.01
25 °C/60%RH 12 months in blister packaging	<0.10	0.06	<0.10	0.06
30 °C/75%RH 12 months in blister packaging	<0.10	0.12	<0.10	0.12
40 °C/75%RH 6 months in blister packaging	<0.10	0.28	<0.10	0.28

## Data Availability

The data presented in this study are available on request from the corresponding author.

## Supplementary Materials

The following supporting information can be downloaded at: https://www.mdpi.com/article/10.3390/pharmaceutics17121627/s1, Figure S1: Powder X-ray diffraction (PXRD) patterns of S-892216 drug substance (Lot D), Figure S2: Plasma concentration profile of S-892216 in rats after a single intravenous administration of S-892216 anhydrate crystal (0.1 mg/kg), Figure S3: Plasma concentration profiles of S-892216 in dogs after a single intravenous administration of S-892216 anhydrate crystal (0.1 mg/kg). Each symbol represents the mean ± SD of 4 dogs, Figure S4: Dissolution testing in FeSSIF-V2 medium (n = 1), Table S1: Gradient program used for HPLC analysis in the rat pharmacokinetic study, Table S2: Gradient program used in HPLC analysis (oral administration), Table S3: Gradient program used in HPLC analysis (intravenous administration), Table S4: Pharmacokinetic parameters of S-892216 after a single intravenous administration of S-892216 anhydrate crystal (0.1 mg/kg) in rats, Table S5: Pharmacokinetic parameters of S-892216 after a single intravenous administration of S-892216 anhydrate crystal (0.1 mg/kg) in dogs, Table S6: Gradient program used in HPLC analysis, Table S7: Solubility of S-892216 drug substance in various solvents, Table S8: Component and composition of solution filled capsules with different antioxidants, Table S9: Degradation products level of oral solution formulations after 6 days of storage, Table S10: Individual pharmacokinetic parameters of S-892216 in rats after a single oral administration of PEG 400 solution (1 mg/kg), Table S11: Individual pharmacokinetic parameters of S-892216 in rats after a single oral administration of the solid dispersion suspension (1 mg/kg), Table S12: Solid dispersion tablets for photostability improvement, Table S13: Results of photostability study. References can be found [55,56,57,58,59,60,61,62].

## Abbreviations

The following abbreviations are used in this manuscript:

COVID-19	Coronavirus disease 2019
SARS-CoV-2	Severe acute respiratory syndrome coronavirus 2
BA	Bioavailability
ADME	Absorption, distribution, metabolism, and excretion
PXRD	Powder X-ray diffraction
PEG 400	Polyethylene glycol 400
PG	Propylene glycol
PVPVA	Polyvinylpyrrolidone-vinyl acetate
HPMCAS	Hydroxypropyl methylcellulose acetate succinate
C_max_	Maximum plasma concentration
T_max_	Time to maximum plasma concentration
AUC	Area under the plasma concentration–time curve
SAD	Single ascending dose
