# Supplementary material for: Selection of Solubility Enhancement Technologies for S-892216, a Novel COVID-19 Drug Candidate
Source: Pharmaceutics. 2025 Dec 18;17(12):1627. doi: 10.3390/pharmaceutics17121627 (PMC12737236; doi:10.3390/pharmaceutics17121627)
Supplement: Supplementary file 1 [file pharmaceutics-17-01627-s001.zip › pharmaceutics-3969577-supplementary.pdf]

Supplementary Materials

Selection of Solubility Enhancement Technologies for S-892216, a Novel COVID-19 Drug Candidate

Ryo Ohashi [1,*], Shuichi Otake [1], Tatsuhiko Murata [1], Ryosuke Watari [2], Shinpei Yoshida [2], Mikiko Kitade [2], Daisuke Kondo [1] and Go Kimura [1]

[1] Formulation R&D Laboratory, Pharmaceutical Technology Research Division, Shionogi & Co., Ltd.,
1-3 Kuise, Terajima 2-chome, Amagasaki 660-0813, Japan;
shuichi.otake@shionogi.co.jp (S.O.);
tatsuhiko.murata@shionogi.co.jp (T.M.); daisuke.kondo@shionogi.co.jp (D.K.);
go.kimura@shionogi.co.jp (G.K.)

[2] Laboratory for Drug Discovery and Development, Shionogi & Co., Ltd., 1-1 Futaba-cho 3-chome,
Toyonaka 561-0825, Japan; ryosuke.watari@shionogi.co.jp (R.W.);
shinpei.yoshida@shionogi.co.jp (S.Y.); mikiko.kitade@shionogi.co.jp (M.K.)

* Correspondence: ryo.ohashi@shionogi.co.jp; Tel.: +81-70-7812-7908

S.1. Materials

S-892216 was synthesized as an anhydrous crystal, and its powder X-ray diffraction (PXRD) pattern of Lot D is presented in Figure S1. Three lots (Lot A, B and C) of the drug substance were used in this study. Lots of A, B, and C used in formulation studies were manufactured by the same synthetic process and share the same crystal form as Lot D. Lot D was not used in formulation development but is shown here as a representative example of the crystal form.

The particle size and purity are as follows:

Lot A: D10 0.78 μm, D50 2.09 μm, D90 4.77 μm, Purity: 95.9%.

Lot B: D10 0.86 μm, D50 3.29 μm, D90 10.15 μm, Purity: 101.7%.

Lot C: D10 0.7 μm, D50 2.7 μm, D90 8.8 μm, Purity: 99.7%.

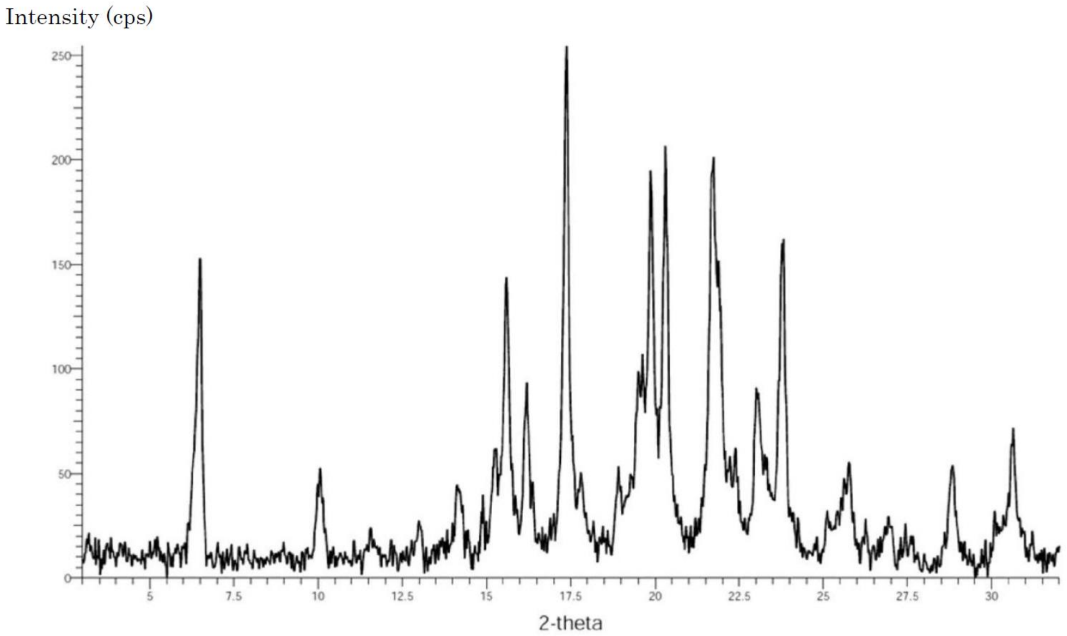

No.	Position (2θ)	Intensity
1	6.5	177.0
2	10.1	76.8
3	13.0	51.6
4	14.1	68.8
5	15.3	85.0
6	15.6	168.1
7	16.2	117.8
8	17.4	278.6
9	18.9	77.6

No.	Position (2θ)	Intensity
10	19.9	219.1
11	20.3	230.8
12	21.7	218.4
13	23.0	115.1
14	23.8	186.3
15	25.8	79.7
16	28.8	78.1
17	30.6	96.0

Figure S1. Powder X-ray diffraction (PXRD) patterns of S-892216 drug substance (Lot D).

S.2. Analytical method for rat pharmacokinetic studies

Table S1. Gradient program used for HPLC analysis in the rat pharmacokinetic study.

Minutes	0.1% formic acid (%)	Acetonitrile (%)
0–1.3	55→30	45→70
1.3–1.31	30→5	70→95
1.31–1.80	5	95
1.80–1.81	5→55	95→45
1.81–2.1	55	45

Table S2. Gradient program used in HPLC analysis (oral administration).

Minutes	0.1% formic acid (%)	Acetonitrile (%)
0–1.3	60→30	40→70
1.3–1.31	30→5	70→95
1.31–1.5	5	95
1.5–1.51	5→55	95→45
1.51–1.8	55	45

Table S3. Gradient program used in HPLC analysis (intravenous administration).

Minutes	0.1% formic acid (%)	Acetonitrile (%)
0–1.3	60→40	40→60
1.3–1.31	40→5	60→95
1.31–1.5	5	95
1.5–1.51	5→55	95→45
1.51–1.8	55	45

S.3. Rat pharmacokinetic study (intravenous administration)

S-892216 anhydrous crystal was dissolved in N, N-dimethyl acetamide/ethanol/20% hydroxypropyl-β-cyclodextrin in carbonate buffer (pH 9.0) (2:3:5 by volume) and intravenously administered to rats (n=2) at 0.1 mg/2 mL/kg under a non-fasted condition. Blood samples were serially collected from the jugular vein up to 24 h after dosing. The total clearance (CL_{tot}), area under the plasma concentration–time curve (AUC_{inf}), elimination half-life ($t_{1/2}$), and distribution volume in the steady state (Vd_{ss}) were calculated by non-compartmental analysis.

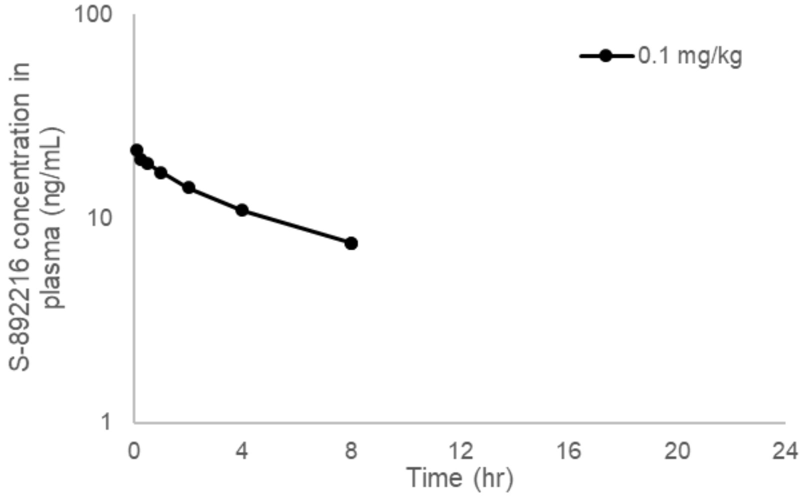

Figure S2. Plasma concentration profile of S-892216 after a single intravenous administration of S-892216 anhydrate crystal (0.1 mg/kg).

Table S4. Pharmacokinetic parameters of S-892216 after a single intravenous administration of S-892216 anhydrate crystal (0.1 mg/kg) in rats.

Dose (mg/kg)	AUC_{inf} (ng·h/mL)	CL_{tot} (mL/min/kg)	$t_{1/2}$ (h)	Vd_{ss} (L/kg)
0.1	179	10.0	7.09	5.56

Data represent the mean of 2 rats.

S.4. Dog pharmacokinetic study (intravenous administration)

For intravenous administration, S-892216 anhydrous crystal was dissolved in dimethyl acetamide/ethanol/20% hydroxypropyl-β-cyclodextrin in carbonate buffer (pH 9.0) (2:3:5 by volume) and intravenously administered to dogs (n=4) at 0.1 mg/0.2 mL/kg under the non-fasted condition. Blood samples were serially collected from the forelimb vein up to 24 h after dosing. The total clearance (CL_{tot}), area under the plasma concentration–time curve (AUC_{inf}), elimination half-life ($t_{1/2}$), and distribution volume in the steady state (Vd_{ss}) were calculated by non-compartmental analysis.

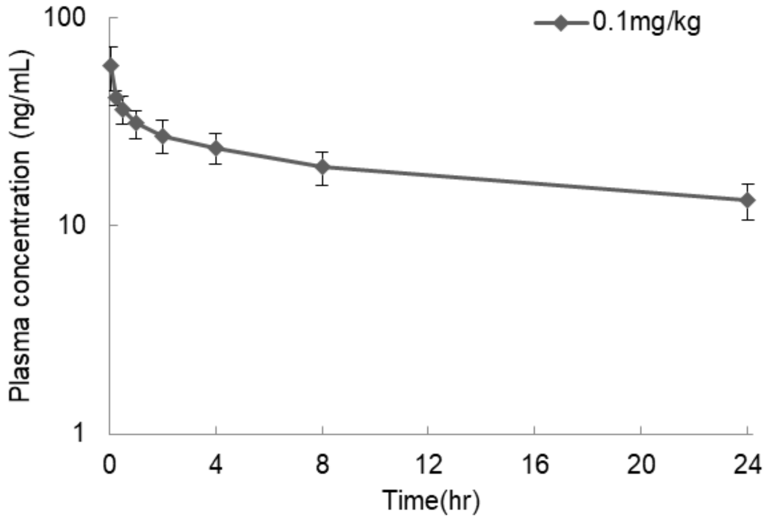

Figure S3. Plasma concentration profiles of S-892216 in dogs after a single intravenous administration of S-892216 anhydrate crystal (0.1 mg/kg). Each symbol represents the mean ± SD of 4 dogs.

Table S5. Pharmacokinetic parameters of S-892216 after a single intravenous administration of S-892216 anhydrate crystallin (0.1 mg/kg) in dogs.

Dose (mg/kg)	AUC_{inf} (ng·h/mL)	CL_{tot} (mL/min/kg)	$t_{1/2}$ (h)	Vd_{ss} (L/kg)
0.1	975±216	1.79±0.48	26.3±4.7	3.86±0.58

Data represent the mean ± SD of 4 dogs.

S.5. Analytical method

Table S6. Gradient program used in HPLC analysis.

Minutes	0.1% formic acid (%)	Acetonitrile (%)
0–27	90→10	10→90
27–30	10	90
30–30.1	10→90	90→10
30.1–39.5	90	10

S.6. Solvent screening for S-892216 anhydrous crystal

A representative example of oral solutions is the self-emulsifying formulation of cyclosporine. This cyclosporine formulation is sold as a soft capsule and oral solution containing 10–100mg of cyclosporine dissolved in a liquid. Because the concentration of the high-dose formulation of cyclosporine mentioned above is 100 mg/mL [55], a similar solubility would be required for oral solution development.

For solvent screening, S-892216 was added to various solvents to a predetermined concentration, and the suspension was stirred at room temperature for 24 h using a stirrer. The solubility was evaluated by observing the state of the solution.

Table S7. Solubility of S-892216 drug substance in various solvents.

Trade name	Source	Solubility (mg/g)
Kolliphor® PS80	BASF, Germany	5–10
Kolliphor® EL	BASF, Germany	5–10
Kollisolv® PEG 400	BASF, Germany	15–20
Capmul® MCM	ABITEC Corporation, USA	<5
Kollisolv® PG	BASF, Germany	<5
Ethanol	FUJIFILM Wako Chemicals, Japan	<5
Glycerine	FUJIFILM Wako Chemicals, Japan	<5
Capryol® 90	Gattefossé, France	<5
Lauroglycol™ 90	Gattefossé, France	<5
Captex® 300 Low C6	ABITEC Corporation, USA	<5

S.7. Improving the chemical stability of the oral solution

When we evaluated the degradation products in a gelatin capsule filled with a PEG 400 oral solution of S-892216, we found a maximum of 0.19% degradation products at time zero, and the level of related substances tended to increase with storage time. These degradation products likely arise from the decomposition of the formulation induced by peroxides, aldehydes, or organic acids present in PEG 400 [56]. Therefore, to prevent degradation, we added an antioxidant to the oral solution. We evaluated the stability of the oral solution containing L-ascorbic acid, citric acid, and α-tocopherol polyethylene glycol succinate (TPGS), which all dissolved in PEG 400. The formulation used in this study is shown in Table S8. We also added PVPVA as a polymer to prevent precipitation.

The levels of degradation products are shown in Table S9. There was a significant suppression in the generation of degradation products when L-ascorbic acid and citric acid were added. Although there are several studies demonstrating the effectiveness of antioxidants in inhibiting drug decomposition in solutions [57] to our knowledge, no research has been reported in which antioxidants are dissolved in the non-aqueous solvent of PEG 400 to inhibit drug decomposition. We found that highly water-soluble antioxidants such as ascorbic acid and citric acid are suitable antioxidants for oral solutions.

Table S8. Component and composition of solution filled capsules with different antioxidants.

Component	Composition			
Formulation	A	B	C	D
S-892216 drug substance (mg)	0.5	0.5	0.5	0.5
PEG 400 (mg)	96.6	95.5	92.2	94.6
PVPVA (mg)	2.9	2.9	2.8	2.8
Ascorbic acid (mg)	-	1.1	-	-
Citric acid (mg)	-	-	4.5	-
TPGS (mg)	-	-	-	2.1
Total (mg)	100.0	100.0	100.0	100.0
Hard gelatin capsule	1 unit	1 unit	1 unit	1 unit

Table 1S9 Degradation products level of oral solution formulations after 6 days of storage.

Formulation		A	B	C	D
Maximum amount of individual degradation product	Time zero	0.19%	N.D.	N.D.	0.17%
	6 days at 25°C/60%RH	0.91%	N.D.	N.D.	0.74%
Total amount of degradation products	Time zero	0.19%	N.D.	N.D.	0.17%
	6 days at 25°C/60%RH	4.38%	N.D.	N.D.	3.45%

N.D.: not detected

S.8. Individual data of Rat Pharmacokinetic Study

Table S10. Individual pharmacokinetic parameters of S-892216 in rats after a single oral administration of PEG 400 solution (1 mg/kg).

Time	Plasma concentration (ng/mL)				
hr	Rat No.1	Rat No.2	Rat No.3	Mean	SD
0.5	199	157	159	172	24
1	187	181	176	181	5
2	157	158	172	162	8
4	110	133	133	125	13
8	81.7	102	104	95.6	12.1
24	10.7	10.4	11.7	10.9	0.7
T_{max} (hr)	0.50	1.00	1.00	0.833	0.289
C_{max} (ng/mL)	199	181	176	186	12
AUC_{inf} (ng·hr/mL)	1794	2028	2092	1972	157
BA (%)	92.5	106	106	101	8

Table S11. Individual pharmacokinetic parameters of S-892216 in rats after a single oral administration of the solid dispersion suspension (1 mg/kg).

Time	Plasma concentration (ng/mL)				
hr	Rat No.4	Rat No.5	Rat No.6	Mean	SD
0.5	143	191	185	173	26
1	167	188	189	181	12
2	139	183	172	165	23
4	103	145	146	131	25
8	64.8	114	114	97.6	28.4
24	4.00	7.20	13.6	8.27	4.89
T_{max} (hr)	1.00	0.50	1.00	0.83	0.29
C_{max} (ng/mL)	167	191	189	182	13
AUC_{inf} (ng·hr/mL)	1420	2190	2290	1970	480
BA (%)	79.3	122	128	110	27

S.9. Dissolution testing

Dissolution testing was performed using the same dissolution apparatus described in Materials and Methods. The dissolution medium was FeSSIF-V2 [58]. Sampling and S-892216 dissolution rate analysis were the same as described in Materials and Methods. The result of dissolution testing is provided in Figure S4.

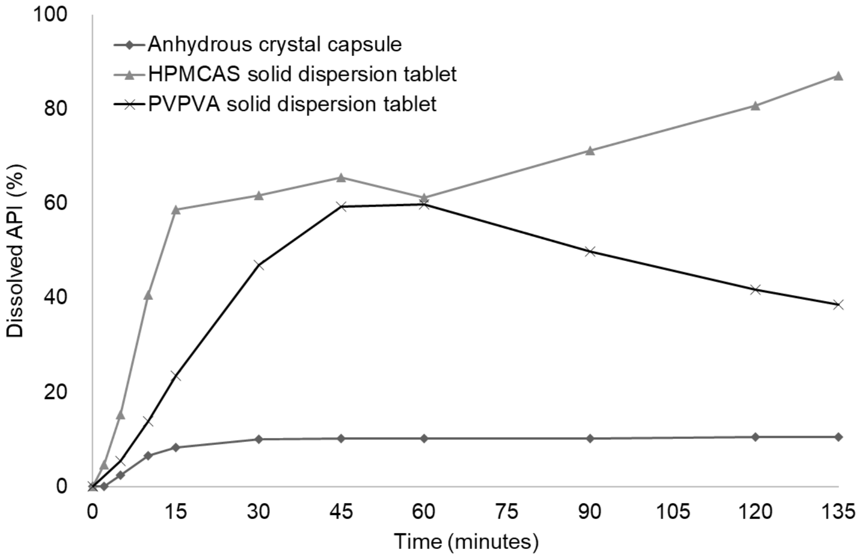

Figure S4. Dissolution testing in FeSSIF-V2 medium (N=1).

S.10. Improvement of photostability

Owing to the photodegradability of S-892216 drug substance, it was necessary to improve its photostability by light protection. Several approaches were considered for improving photostability, including the addition of light-absorbing substances, film coating, and light blocking with primary packaging [59]. Among these options, film coating also improves swallowability and masks the taste, which are expected to improve patient acceptability [60]. Therefore, we attempted to improve photostability by applying a film coating. The component and composition of the uncoated and coated tablets used are shown in Table S12. The manufacturing process, including spray drying, dry granulation, blending, lubrication, tableting, and coating, is described in Section 2.2.5. We aimed to improve light stability by incorporating red ferric oxide and yellow ferric oxide as colorants for light protection. The results of the photostability test are shown in Table S13. We found that degradation upon light exposure was completely suppressed by applying a film coating. Interestingly, we improved photostability without adding titanium dioxide, a conventional light-blocking colorant [61]. ESFA has expressed concern about the genotoxicity of titanium oxide [62]. It is possible that in the future there will be a trend toward reducing the use of titanium oxide in the pharmaceutical industry. This study demonstrated an approach for improving photostability without the addition of titanium oxide. However, Salawi et al. reported that nifedipine and molsidomine solutions degrade by more than 30% within 12 h of photostability testing [60]. Because the photostability of S-892216 solid dispersion tablets is greater than that of these drugs, it is necessary to consider selecting titanium oxide depending on the chemical property of the drug substance.

Table S12. Solid dispersion tablets for photostability improvement.

Component	Composition (mg)	
Formulation	Uncoated tablets	Coated tablets
S-892216-PVPVA Solid dispersion	160.0	160.0
(as S-892216)	(40.0)	(40.0)
Mannitol	148.8	148.8
Microcrystalline cellulose	37.2	37.2
Croscarmellose sodium	40.0	40.0
Colloidal silicon dioxide	2.0	2.0
Sodium stearyl fumarate	12.0	12.0
Sub-total	400.0	400.0
Coating agent		16.0
Hypromellose		(70.330% w/w)
Talc	-	(28.966% w/w)
Red Ferric Oxide		(0.352% w/w)
Yellow Ferric Oxide		(0.352% w/w)

Total	416.0

Table S13. Results of photostability study.

	Total degradation products (%)		
Storage condition and period	Time zero	25 °C/60% RH, D65 lamp, 120 million lux·hr	25 °C/60% RH, sealed D65 lamp, 120 million lux·hr
Uncoated tablets	0.20	3.59	0.20
Coated tablets	0.19	0.55	0.18
